# Whole-Genome Sequencing and Comparative Genome Analysis Provided Insight into the Predatory Features and Genetic Diversity of Two *Bdellovibrio* Species Isolated from Soil

**DOI:** 10.1155/2018/9402073

**Published:** 2018-04-10

**Authors:** Omotayo Opemipo Oyedara, Aldo Segura-Cabrera, Xianwu Guo, Temidayo Oluyomi Elufisan, Rafael Alejandro Cantú González, Mario A. Rodríguez Pérez

**Affiliations:** ^1^Instituto Politécnico Nacional, Centro de Biotecnología Genómica, 88710 Reynosa, TAMPS, Mexico; ^2^Department of Biological Sciences, College of Science, Engineering and Technology, Faculty of Basic and Applied Science, Osun State University, PMB 4494, Osogbo, Osun State, Nigeria; ^3^Red de Estudios Moleculares Avanzados, Instituto de Ecología, A.C., Xalapa Enriquez, VER, Mexico; ^4^National Center for Technology Management, Agency of the Federal Ministry of Science and Technology (FMST), Obafemi Awolowo University, Ile-Ife, Nigeria

## Abstract

*Bdellovibrio* spp. are predatory bacteria with great potential as antimicrobial agents. Studies have shown that members of the genus *Bdellovibrio* exhibit peculiar characteristics that influence their ecological adaptations. In this study, whole genomes of two different *Bdellovibrio* spp. designated SKB1291214 and SSB218315 isolated from soil were sequenced. The core genes shared by all the *Bdellovibrio* spp. considered for the pangenome analysis including the epibiotic *B. exovorus* were 795. The number of unique genes identified in *Bdellovibrio* spp. SKB1291214, SSB218315, W, and *B. exovorus* JJS was 1343, 113, 857, and 1572, respectively. These unique genes encode hydrolytic, chemotaxis, and transporter proteins which might be useful for predation in the *Bdellovibrio* strains. Furthermore, the two *Bdellovibrio* strains exhibited differences based on the % GC content, amino acid identity, and 16S rRNA gene sequence. The 16S rRNA gene sequence of *Bdellovibrio* sp. SKB1291214 shared 99% identity with that of an uncultured *Bdellovibrio* sp. clone 12L 106 (a pairwise distance of 0.008) and 95–97% identity (a pairwise distance of 0.043) with that of other culturable terrestrial *Bdellovibrio* spp., including strain SSB218315. In *Bdellovibrio* sp. SKB1291214, 174 bp sequence was inserted at the host interaction *(hit)* locus region usually attributed to prey attachment, invasion, and development of host independent *Bdellovibrio* phenotypes. Also, a gene equivalent to *Bd0108* in *B. bacteriovorus* HD100 was not conserved in *Bdellovibrio* sp. SKB1291214. The results of this study provided information on the genetic characteristics and diversity of the genus *Bdellovibrio* that can contribute to their successful applications as a biocontrol agent.

## 1. Background

Studies on predatory bacteria have received much attention recently because of the possibility to harness their potentials for the biocontrol of pathogenic bacteria. *Bdellovibrio* spp. are versatile predatory bacteria that specialize in preying upon a wide range of Gram-negative bacteria, utilizing the resulting molecules from their attack for growth and reproduction [1]. Based on the mechanism of predation, there are two species of the genus *Bdellovibrio*, namely, *B. bacteriovorus* and *B. exovorus*. The former invade the periplasmic space of its prey while the latter attaches to the external surface (epibiotic) to derive its nutrients [2, 3]. Members of the genus *Bdellovibrio* are diverse with some of them exhibiting unique features that can influence their ecological adaptations. For instance, *B. bacteriovorus* strain W has the unique ability to develop a dormant structure called bdellocyst which can help them survive unfavorable conditions [4]. *B. bacteriovorus* is an obligate predatory bacterium. However, a strain isolated from Tiber River (*B. bacteriovorus* strain Tiberius) has shown the unique ability to grow simultaneously in the presence and absence of prey [5]. *B. bacteriovorus* strains that replicate and grow on nutrient-rich media without bacterial prey, usually called host-independent (HI) phenotypes often have mutations at a region of their genomes known as host interaction *(hit)* locus, tagged gene *Bd0108* in *B. bacteriovorus* HD100. The *hit* locus has been proposed to regulate the formation of type IV pilus needed for prey attachment and invasion [6].

Ancient and recent lateral gene transfers have been reported to occur in *Bdellovibrio* spp., and this may play a crucial role in their evolution probably leading to the development of unique features that can impact on their predatory lifestyle [5, 7, 8]. Thus, whole-genome sequence analysis can provide an in-depth understanding of variations in predation traits and evolution of *Bdellovibrio* spp. in turns helping in their successful application as biocontrol agents against bacterial pathogens. For instance, acquisition of pathogenic islands and alteration in their genomic structure via horizontal gene transfer may have an impact that can influence their application as biocontrol agents.

*Bdellovibrio* spp. found in soil are heterogeneous with different populations coexisting in the soil [9]. In our previous study, we isolated two different strains of *Bdellovibrio* spp. designated SKB1291214 and SSB218315 from soil samples in the same environment. The strains exhibited different phenotypes based on the time required to form plaque on Gram-negative bacteria prey lawns and prey range which was limited to some members of the family Enterobacteriaceae in *Bdellovibrio* sp. SKB1291214 [10]. Furthermore, the amplification of host interaction *(hit)* locus in *Bdellovibrio* sp. SKB1291214 using the PCR technique was unsuccessful. Therefore, we use whole-genome sequencing and comparative genomics as a tool to understand the genetic variations between these two strains and determine their relatedness with other reported genomes retrieved from the NCBI database.

## 2. Materials and Methods

### 2.1. Bacterial Strains and Genome Sequencing

*Bdellovibrio* spp. strains SKB1291214 and SSB218315 were isolated from soil samples obtained from different locations on a plot of land (26.069678N′, −98.313108W′ and 26.069446N′, −98.312902W′) within the Center for Genomic Biotechnology, National Polytechnic Institute (IPN as in Spanish) located in the city of Reynosa, Mexico. The *Bdellovibrio* spp. were cultured as described in our earlier report [10]. The genomic DNA (gDNA) was extracted using the Wizard® Genomic DNA Purification Kit (Madison, Wisconsin, USA) according to the manufacturer's instructions. The gDNA was subjected to optical density measurements in NanoDrop and Qubit (Thermo Fisher Scientific, Waltham, MA, USA). DNA migration in agarose gel electrophoresis was done to confirm the purity and concentration prior to fragmentation in Bioruptor (Diagenode Inc., Denville, NJ, USA). Fragmented gDNA was tested for size distribution and concentration using a 2200 Tapestation (Agilent Technologies Inc., Santa Clara, CA, USA) and subjected to Illumina library preparation using the Beckman SPRI-TE automated liquid handler and library prep reagents (Beckman Coulter, CA, USA). The resulting library was tested for size distribution and concentration by 2200, NanoDrop, and Qubit. The libraries were then loaded for Illumina NextSeq sequencing according to the standard operation. Paired-end 75 nucleotide (nt) reads were generated and checked for data quality using FASTQC (Babraham Institute, Cambridge, UK).

### 2.2. Genome Assembly and Annotation

The pair-end reads generated from the Illumina sequencing were trimmed using the Sickle tool 1.33 [11], assembled de novo using the SPAdes assembler version 3.10.0 [12], and then arranged into scaffolds using the MeDuSa scaffolder 1.3 [13]. The resulting contigs were then improved using Iterative Mapping and Assembly for Gap Elimination (IMAGE) [14]. Quast software was used to assess the quality of the generated scaffold based on the number of contigs and the N50 [15]. The genome sequences were automatically annotated using the NCBI Prokaryotic Genome Annotation Pipeline (PGAP) (https://www.ncbi.nlm.nih.gov/genome/annotation_prok/) and Rapid Annotation using Subsystem Technology (RAST) server [16]. Prophage sequences and genomic islands were predicted from the genomes using PHASTER [17] and IslandViewer4 [18] online application, respectively.

### 2.3. Phylogenetic Tree Construction and Estimation of Pairwise Evolutionary Divergence between 16S rRNA Gene Sequences

The 16S rRNA gene sequences were aligned using the MUSCLE alignment tool with default parameters, and a phylogenetic tree was constructed using the maximum likelihood method based on the Kimura 2-parameter model. Bootstrap values were calculated to test the robustness of interior node support and were obtained by conducting 1000 pseudoreplicates using MEGA© 6.0 software [19]. Pairwise evolutionary divergence (distance) was conducted in MEGA© 6.0 software using the Kimura 2-parameter model with 1000 bootstrap replications.

### 2.4. Comparative Genome Analysis

For the whole-genome comparative study, genomes of eight *Bdellovibrio* spp. were retrieved from the NCBI database and compared with the genomes of the study *Bdellovibrio* strains (*Bdellovibrio* sp. SKB1291214 and *B. bacteriovorus* SSB218315). The retrieved genomes include that of the epibiotic *B. exovorus* JSS (NC_020813), *B. bacteriovorus* strains HD100 (NC_005363), W (NZ_CP002190), Tiberius (NC_019567), 109J (NZ_CP007656), R0 (LUKE00000000), EC13 (LUKD00000000), and BER2 (LUKF00000000).

The similarity among the genomes based on average amino acid identity (AAI) was inferred using the ANI/AAI-Matrix Genome-based distance matrix calculator [20]. A pangenome analysis was carried out with the bacterial pangenome analysis (BPGA) tool [21] using the two study genomes and genomes of five reported *Bdellovibrio* spp. These include *B. exovorus* JSS (NC_020813) and *B. bacteriovorus* strains HD100 (NC_005363), W (NZ_CP002190), Tiberius (NC_019567), and 109J (NZ_CP007656). BLASTP search and functional annotation analysis of the core and unique genes were done with the BLAST2GO pipeline [22] using the default settings with the BLAST expectation value (*E* value) of 1.0*E* − 3. The *hit* locus regions of *Bdellovibrio* spp. HD100, SKB1291214, and SSB218315 were compared by constructing a genome map using the KBase online software (https://kbase.us/), followed by BLASTP analysis of the *hit* regions in ExPASy Bioinformatics Resource Portal (https://www.expasy.org/). Alignment of the regions corresponding to the *hit* locus in the different *Bdellovibrio* strains was done using the multiple sequence alignment tool, Clustal Omega [23].

### 2.5. Nucleotide Sequence Accession Numbers

The whole-genome shotgun project has been deposited at DDBJ/ENA/GenBank databases under the accession NELQ00000000 for *Bdellovibrio* sp. SKB1291214 (the version described in this paper is version NELQ01000000). The complete genome sequence of *B. bacteriovorus* SSB218315 was deposited in the same databases under accession number CP020946.

## 3. Result and Discussion

### 3.1. Genomic Features of *Bdellovibrio* spp. Strains SKB1291214 and SSB218315

The genomic features of *B. bacteriovorus* strains SSB218315 and *Bdellovibrio* sp. strain SKB1291214 are summarized in Table 1. The genome size of *B. bacteriovorus* SSB218315 and *Bdellovibrio* sp. SKB1291214 is 3,769,537 bp and 3,730,590 bp, respectively. *Bdellovibrio* spp. are small but have large genomes (approximately 3.7 Mb) that encode predation factors presumed important to seek and lyse prey cells [24]. The percentage GC content in *Bdellovibrio* sp. SKB1291214 (44.8%) is low compared to *B. bacteriovorus* SSB218315 (50.5%). Lambert et al. [25] reported some genes expressed during predation in *B. bacteriovorus* HD100. These include genes that are up- and downregulated at the early stage of *B. bacteriovorus* HD100 (30 minutes) infection as it switches from the motile prey-seeking attack stage to the intraperiplasmic phase, when it establishes itself in the prey cell. And because of the phenotypic differences observed between *Bdellovibrio* spp. SKB1291214 and SSB218315 [10], genome analysis was done to identify and compare the gene equivalent described by Lambert et al. [25] in the study strains. From BLASTP analysis results, *B. bacteriovorus* SSB218315 have all the described gene equivalent (Additional file 1a). However, among the 75 described upregulated genes, *Bd1230 (lamb)*, *Bd0487*, and *Bd2298* equivalents in *B. bacteriovorus* HD100 were absent in the genome of *Bdellovibrio* sp. SKB1291214. The *Bd1230 (lamb)* gene encodes maltoporin, an outer membrane protein that is important for sugar transport in Gram-negative bacteria, and it is usually expressed when *Bdellovibrio* degrades its prey. The genes *Bd0487* and *Bd2298* are found only in the genome of *Bdellovibrio*, and they are significantly upregulated when *Bdellovibrio* enters the periplasmic phase of growth [25]. Furthermore, eight out of the forty-one reported downregulated gene equivalents implicated in the attack phase of *Bdellovibrio* were absent in the genome of SKB1291214. These genes include the equivalent of *Bd3260*, *Bd2608*, *Bd2400*, *Bd0737*, and *Bd0992 (cwlJ)* encoding putative membrane proteins and enzymes (putative lipase and cell wall hydrolase) that play a role in prey attachment and penetration. Two gene equivalents *Bd0880* and *Bd0931* encoding stress response proteins, a homologue of periplasmic adaptor protein CpxP and transcriptional regulator, and MerR family were also absent in the genome of *Bdellovibrio* sp. SKB1291214. *B. bacteriovorus* uses type IV pilus to attach and subsequently invade prey cells. The gene equivalent of *Bd0108* which encode proteins that function in regulating type IV pilus secretion in *Bdellovibrio* was also not present in the genome of SKB1291214. The missing gene equivalents described above might be playing important roles during *Bdellovibrio* predation. And thus, the absence of these genes in the genomes of *Bdellovibrio* sp. SKB1291214 can affect its rate of predation.

The RAST annotation server also predicted some genes presumed to enhance predation in *Bdellovibrio* spp. (Additional files 1b–f). These include genes encoding motility and chemotaxis factors, transport system including type IV pilus, stress response proteins, degradative proteins, and siderophores, and other defense factors.

The rapid motility of *Bdellovibrio* helps in prey location [26]. From RAST annotation and manual curation, about 75 genes encoding motility and chemotaxis factors were identified in the study *Bdellovibrio* strains. Among these factors are five adventurous gliding motility factors R, S, T, U, V, and *MglA* used by *Bdellovibrio* spp. to glide on solid surfaces and find prey in environments with a low water content such as biofilms [27, 28]. The RAST annotation server also predicted a sequence called diguanylate cyclase/phosphodiesterase (GGDEF and EAL domains) with PAS/PAC sensor(s) in *Bdellovibrio* spp. SKB1291214 (B9G69_13450, B9G69_14735, B9G69_01735, and B9G69_08345) and SSB218315 (B9G79_16530, B9G79_14755, B9G79_11600, B9G79_00860, and B9G79_03750) as stress response proteins. Proteins that possess this GGDEF sequence secrete cyclic di-GMP, a signalling protein that controls *Bdellovibrio* to grow either as a predator that require prey for survival or a host-independent phenotype that can replicate on nutrient-rich medium. Four enzymatically competent GGDEF protein domains designated DgcA, DgcB, DgcC, and DgcD have been reported [29]. In the study of Hobley et al. [29], Δ*dgcA* mutants became a nonmotile host-independent strain that can grow axenically on nutrient-rich medium only. The ΔdgcA mutants can invade, replicate, and septate inside prey cells but cannot glide out of the prey cells to look for new prey. The Δ*dgcB* mutants became flagellated host-independent strains. The Δ*dgcC* mutants developed into predatory strains that are not capable of growing as host-independent (HI) strains. However, for ΔdgcC to grow axenically, they require additional or secondary mutation. Mutation of the *dgcD* gene did not result in any phenotypic alteration with mutants Δ*dgcD* growing both as host-dependent (HD) and HI. BLASTP analysis showed that GGDEF protein domains DgcA, DgcB, and DgcC are conserved in the two *Bdellovibrio* strains. However, the DgcD is not conserved in *Bdellovibrio* sp. SKB1291214, a similar result observed in *B. bacteriovorus* Tiberius [29].

*Bdellovibrio* spp. have been described to be nonpathogenic to human [30]. However, genomes of *Bdellovibrio* spp. SKB129124 and SSB218315 and other *Bdellovibrio* strains encode genes annotated as collagenase and hemolysin, virulence factors associated with some pathogens of human such as *Staphylococcus aureus* [31] and *Vibrio vulnificus* [32]. The genome of *B. bacteriovorus* SSB218315 also encodes genes annotated as RTX toxins, a factor that has different biological functions such as pore-forming leukotoxin, metalloprotease, and lipase activities [33]. BLAST2GO software was used to carry out a BLASTP search and assign gene ontology (GO) to the gene products of the sequences annotated as hemolysin, collagenase, and RTX toxins (Additional file 1g). The GO of the sequences annotated as hemolysin III is cytolysis, and the BLASTP analysis revealed a conserved hemolysin III-related protein domain. The BLASTP analysis of the annotated collagenase revealed a conserved U32 family peptidase protein domain. However, collagenase belongs to the U32 family peptidase [34]. Identification of the biological roles of hemolysin III and collagenase in *Bdellovibrio* spp. will aid in their successful application as biocontrol agents against human pathogens. Analysis using the Pfam database [35] revealed that the RTX-toxin sequences did not have toxin domain but rather a protein domain identified as a regulator of chromosome condensation (RCC1) repeat. Thus, the annotated RTX toxin might be performing a different role than being involved in toxin production.

Bacteria can acquire genomic islands (GEIs) via horizontal gene transfer (HGT). These GEIs can confer adaptive features such as antibiotic resistance, survival features, and metabolic activities, metabolism of complex compounds on the bacteria [36]. Some distinguishing features of GEIs include association with genes encoding tRNA, integrase, or transposase, and possession of the percentage G + C content that is different from another part of the genome [37]. Predicted GEIs of *Bdellovibrio* strains SKB1291214 and SSB218315 include hypothetical proteins, peptidase, septation protein spoVG (in *B. bacteriovorus* SSB218315), and survival protein *surA* which can aid the survival of bacteria at the stationary growth phase (Additional file 2).

### 3.2. Phylogeny and Amino Acid Identity of *Bdellovibrio* Species

**Figure 1:**
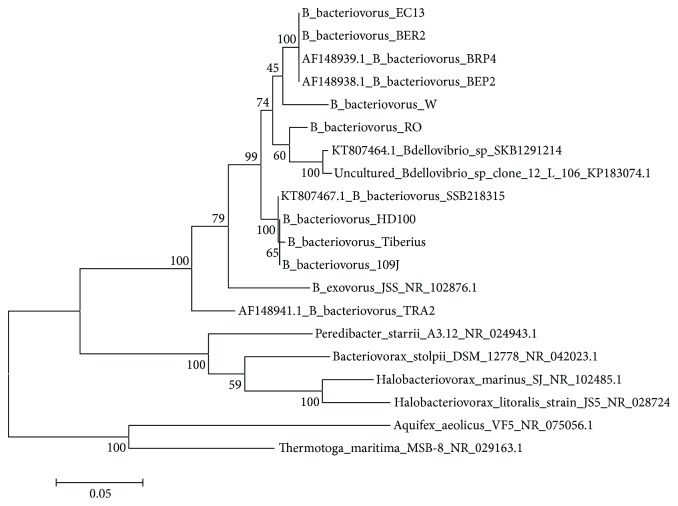
Molecular phylogenetic analysis by a maximum likelihood method using 16S rRNA gene sequences. The evolutionary history was inferred by using the maximum likelihood method based on the Kimura 2-parameter model with 1000 bootstrap replications. The percentage of trees in which the associated taxa clustered together is shown next to the branches. Initial tree for the heuristic search was obtained automatically by applying neighbor-joining and BioNJ algorithms to a matrix of pairwise distances estimated using the maximum composite likelihood (MCL) approach and then selecting the topology with the superior log likelihood value. The analysis involved 20 16S rRNA gene nucleotide sequences. Evolutionary analyses were conducted in MEGA6 [19].

**Figure 2:**
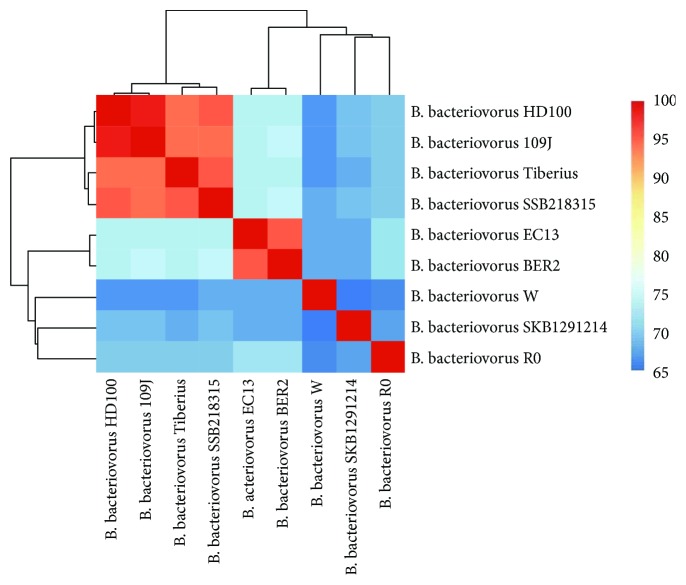
The average amino acid identity matrix clustering analysis of the whole genomes of nine *Bdellovibrio* strains. Colours represent bands of percent identity. The heatmap was generated in R package plots using heatmap.2 function.

The phylogenetic analysis was done to compare the 16S rRNA gene sequences of *Bdellovibrio* sp. SKB1291214 and *B. bacteriovorus* SSB218315 with sequences of other members of the genus *Bdellovibrio* and their relatives that belong to the genus *Bacteriovorax*, *Peredibacter*, and *Halobacteriovorax* (Figure 1). The 16S rRNA sequence of strains SKB1291214 and SSB218315 showed 96% similarity with a pairwise evolutionary distance of 0.043 (Additional file 3). The strain SKB1291214 shared 99% identity with an uncultured *Bdellovibrio* sp. clone12 L 106 (pairwise distance of 0.008) while strain SSB218315 shared 100% identity with other culturable terrestrial *B. bacteriovorus* which include *B. bacteriovorus* strain HD100 (pairwise distance 0.001) and Tiberius (pairwise distance 0.004). The phylogenetic tree showed that the two *Bdellovibrio* strains SKB1291214 and SSB218315 are phylogenetically different despite being isolated from soil samples in the same environment. Further species delineation was done to examine the AAI among the *Bdellovibrio* strains. For strains to belong to the same species, they must have ANI and AAI ≥ 95%, <10 Karlin genomic signature, and >70% in silico GGDH [38]. The AAI between strain SKB1291214 and other strains was very low (63.70–67.68%) while strain SSB218315 shared a high AAI value of 95% with *B. bacteriovorus* strains HD100, Tiberius, and 109J (Figure 2). The result showed that strain SSB218315 is closely related to HD100, Tiberius, and 109J and thus, they can conveniently be grouped as the same species. Meanwhile, considering the percentage GC content, phylogenetic tree clustering pattern, and AAI value, strain SKB1291214 could be grouped as a novel species; however, further analysis is needed.

### 3.3. Pangenome Analysis

**Figure 3:**
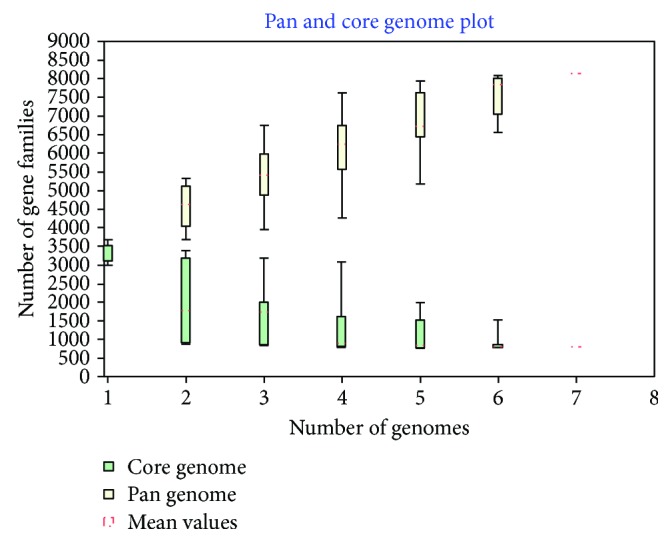
Plot showing the core and pangenomes of *Bdellovibrio* spp. The total number of genes or pangenome (yellow) and shared or core genome (green) for 7 *Bdellovibrio* strains are shown on the plot. The pangenome is made up of 8134 genes while the core genomes are made up of 795 genes.

A bacterial pangenome analysis (BPGA) tool was used to carry out pangenome analysis of eight *Bdellovibrio* spp. The pangenome is made up of 8134 genes, and the *Bdellovibrio* spp. shared 795 genes as core genomes (Figure 3, Additional file 4a). The BGPA predicted the pangenome of *Bdellovibrio* spp. as open based on the power law regression of the program (Additional file 4b). The total number of unique genes found in *Bdellovibrio* spp. SKB1291214 and SSB218315 is 1343 and 113, respectively (Table 2). The epibiotic *B. exovorus* JJS and bdellocyst-forming *B. bacteriovorus* W have a total of 1572 and 857 unique genes, respectively. The GO of the unique genes in *Bdellovibrio* sp. SKB1291214 revealed that they are rich in proteins involved in molecule transport, oxidation-reduction process, signal transduction, hydrolase activity phosphorylation, and nucleotide and ion binding (Additional files 4c–f). *B. exovorus* JJS has the highest number of unique genes (1572), and among these are three genes encoding type II CRISPR-associated endonuclease Cas1, CRISPR-associated Cas2, and type II CRISPR RNA-guided endonuclease Cas9 which usually act to defend prokaryotes against any invading foreign genetic material. These CRISPR genes are however absent in the genome of the periplasmic *Bdellovibrio* spp.

A comparative genomic study by Pasternak et al. [39] identified protein families that are specific to predatory bacteria and differentiate them from the nonpredatory bacteria. All the fifteen protein families reported to be specific to predatory bacteria were present in *Bdellovibrio* spp. SKB1291214 and SSB218315. Homologue of genes encoding two protein families, 4-diphosphocytidyl-2-C-methyl-D-erythritol kinase (B9G69_00295; mean similarity value = 57; *E* value = 1.33*E* − 134) and indole-3-glycerol phosphate synthase (B9G69_04640; mean similarity value = 74; *E* value = 0.0), reported to be specific to nonpredatory bacteria was found among the unique genes of *Bdellovibrio* sp. SKB1291214 (Additional file 4c). Predatory bacteria are different from the nonpredators based on the pathway utilized for the biosynthesis of isoprenoids. While the nonpredators use the deoxy-d-xylulose 5-phosphate (DOXP) or nonmevalonate pathway, the predators use the mevalonate pathway for the biosynthesis of isoprenoids [39]. The 4-diphosphocytidyl-2-C-methyl-D-erythritol kinase is an enzyme required for the nonmevalonate pathway synthesis of isoprenoid, and its presence in *Bdellovibrio* sp. SKB1291214 presumably is a result of horizontal gene transfer (HGT).

One of the differences between *B. exovorus* and *B. bacteriovorus* is the mechanism they use for prey attack [2, 3]. The latter is characterized by the invasion of the prey periplasm while the former are not capable of penetrating into their prey. During prey invasion in *B. bacteriovorus* HD100, three genes tagged *Bd0816*, *Bd3459*, and *Bd3460* play an important role. [26, 40, 41]. The *Bd0816* and *Bd3459* encode D-alanyl-D-alanine carboxypeptidase usually expressed at the point of prey entry while *Bd3460* encodes a protein called ankyrin which protects *Bdellovibrio* hydrolytic enzymes which it secretes during prey invasion.

Genes encoding D-alanyl-D-alanine carboxypeptidase were present among the unique genes of *Bdellovibrio* sp. SKB1291214 (B9G69_09970 and B9G69_09965) and *B. exovorus* JJS (A11Q_2041) (Additional files 4c and d). However, gene encoding ankyrin was absent in the genome of *B. exovorus* JJS but present in the genome *Bdellovibrio* spp. SKB1291214 and SSB218315 which are closer to the *B. bacteriovorus* HD100 compared to the epibiotic *B. exovorus*. While the presence of genes encoding D-alanyl-D-alanine carboxypeptidase is a general feature of the genus *Bdellovibrio* spp., the ankyrin-encoded genes are limited to the periplasmic members of the genus *Bdellovibrio*. The predation mechanism of *B. exovorus* does not require prey invasion, hence, the possible reason why it does not have the gene equivalent of *Bd3460* in its genome. Furthermore, the unique genes of SKB1291214 also contain the Autographivirinae Erwinia phage-associated region coding for protein *AmsF* (B9G69_00395) which is involved in amylovoran biosynthesis. Amylovoran is an exopolysaccharide that plays a role in the pathogenesis of *Erwinia carotovora* [42].

The presence of genes encoding integrases (among predicted GEIs), transposases, phage-associated protein *AmsF*, and nonpredatory bacteria-associated 4-diphosphocytidyl-2-C-methyl-D-erythritol kinase (among the unique genes) suggests the occurrence of HGT in *Bdellovibrio* sp. SKB1291214. From the BLASTP analysis done with the BLAST2GO software using an *E* value threshold of 1*E* − 6, there is an indication that some of the unique genes are acquired horizontally from bacteria that belong to groups other than class Deltaproteobacteria. These groups include the Alphaproteobacteria (B9G69_00040, DUF4334 domain-containing and B9G69_13200, AraC family transcriptional regulator), Betaproteobacteria (B9G69_13210, Com family DNA-binding transcriptional regulator and B9G69_11675, FAD: FMN transferase), Gammaproteobacteria (B9G69_13180, glyoxalase bleomycin resistance dioxygenase; B9G69_13235, terminase small subunit; B9G69_13225, AlpA family transcriptional regulator; and B9G69_13230, bacteriophage), Epsilonproteobacteria (B9G69_11670, nuclease), Bacteroidetes (B9G69_18290, molybdopterin-binding oxidoreductase), and Cyanobacteria (B9G69_17905, HAMP domain-containing and B9G69_00045, 3 and saliva-related transmembrane). This result corroborates the earlier findings of Gophna et al. [7]. A study on the extent and frequency of HGT in *Bdellovibrio* spp. will provide useful information that can aid their successful application as biocontrol agents.

### 3.4. Analysis of the Host Interaction *(hit)* Locus

**Figure 4:**
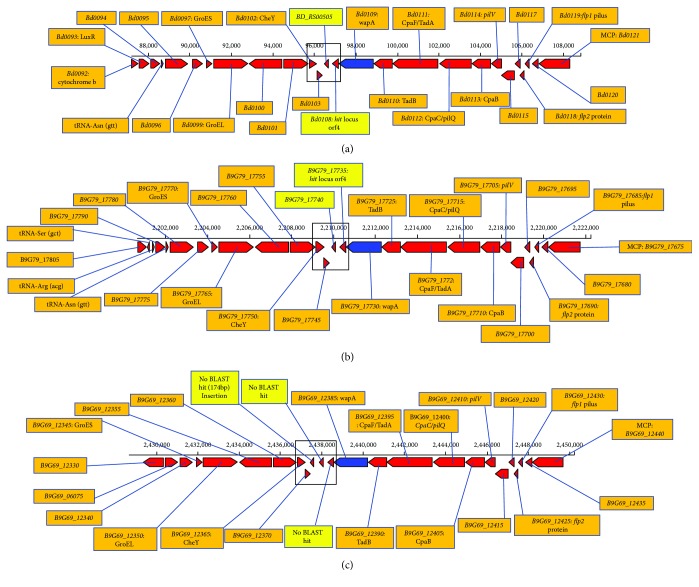
Diagrammatic comparison of the *hit* locus and the adjoining regions between *Bdellovibrio* spp. HD100 (a), SSB218315 (b), and SKB1291214 (c). The major differences can be observed at the region after the *wapA* gene. There was no BLAST hits for gene equivalent of *hit* locus orf4 *(Bd0108)*, *BD_RS00505* (new locus tag assigned to the fragment in *B. bacteriovorus* HD100) and another uncharacterized gene in *Bdellovibrio* sp. SKB1291214. The maps were generated using KBase software (http://biorxiv.org/content/early/2016/12/22/096354), and BLAST analysis was done in ExPASy Bioinformatics Resource Portal (http://www.expasy.org/). *CpaB*, *CpaF/TadA*, *TadB*, *pilQ/Cpac*, *pilV*, *flp1*, *flp2* (genes associated with type IV pilus secretion), chemotaxis protein (MCP: methyl accepting chemotaxis protein, chemotaxis protein *CheY*), heat-shock protein (*GroES* and *GroEL*), cell wall-associated protein *(wapA)*, host interaction *(hit)* locus orf.

*B. bacteriovorus* has been described to have the ability to switch from being predatory usually referred to as host dependent (HD) to growing on nutrient-rich medium axenically, sometimes referred to as host independent (HI). Mutation at a region identified as host interaction *(hit)* locus has been reported to be responsible for the conversion from the HD to HI phenotypes. The *hit* locus has been described to be made up of an open reading frame (ORF) tagged *Bd0108* and part of ORF tagged *Bd0109* encoding a putative cell wall-associated protein in *B. bacteriovorus* HD100 [6]. There are *pil* genes located upstream of the *hit* locus (Figure 4(a)). These *pil* genes encode structural proteins for the formation of the type IV pilus system needed for prey adherence and colonization. The genes *Bd0113* and *Bd0114* are responsible for the pilus assembly while the TadA *(Bd0110)* and TadB *(Bd0111)* encode ATPase that provides energy for the type IV pilus secretion [43]. And downstream of the *hit* locus are genes tagged BD_RS00505 (new locus tag for *B. bacteriovorus* HD100 genes) and *Bd0103* in *B. bacteriovorus* HD100; both genes encode hypothetical proteins of unknown function. The above-described genes (the *Bd0108*, *Bd0109*, pil genes, BD_RS00505, and Bd0103) are inserted in between two genes *Bd0102* and *Bd0121* encoding chemotaxis factors. In our previous study, *hit* locus was successfully amplified in *B. bacteriovorus* SSB218315 using the PCR technique. The negative result obtained from the PCR amplification of the *hit* locus in *Bdellovibrio* sp. SKB1291214 made us construct genomic maps to compare the *hit* locus region between *Bdellovibrio* spp. HD100, SSB218315, and SKB1291214 (Figures 4(a)–4(c)). From the result of the BLASTP and multiple sequence alignment analysis, the region corresponding to the *Bd0108* (*hit* locus) is not conserved in *Bdellovibrio* sp. SKB1291214. (Additional file 5). Furthermore, a fragment of 174 bp absent in HD100 and SSB218315 was found inserted between gene equivalent *Bd0102* and *BD_RS00505* in SKB1291214 (Figure 4(b)). This fragment produces an insignificant *E* value with BLASTP analysis. Comparative analysis revealed that the gene equivalent *Bd0109* is conserved among the *Bdellovibrio* spp. including *Bdellovibrio* spp. SKB1291214 and *B. exovorus* JJS. Thus, this suggests that *Bd0109* gene may have an important role in the predatory activities of *Bdellovibrio* spp. Also, variations in the sequence of *Bd0108* may not be sufficient to hinder prey predation *Bdellovibrio* spp. Because *Bdellovibrio* spp. that have a mutation at the *hit* locus can be cultured axenically [6], we attempted to culture *Bdellovibrio* spp. SKB1291214 and SSB218315 on nutrient-rich medium in the absence of prey using three different techniques described by Ferguson et al. [44], Lambert and Sockett [45], and Seidler and Starr [46]. However, we could not successfully isolate the HI phenotypes using the three approaches, though all the yellow bacterial colonies obtained from the method exhibited the phenotypic characteristics described in the previous research.

## 4. Conclusion

Members of the genus *Bdellovibrio* have been reported to have potential applications as biocontrol agents against pathogens. This study focused on the whole-genome sequencing and comparative analysis of two *Bdellovibrio* spp. that showed phenotypic differences. The comparative analysis showed that *B. bacteriovorus* SSB218315 is genetically related to the soil-derived *B. bacteriovorus* HD100. We also observed that the *Bdellovibrio* sp. SKB1291214 is distinctively different from the epibiotic *B. exovorus*; although SKB1291214 showed traits associated with the intraperiplasmic predatory lifestyle, it is still different from SSB218315 and HD100 based on the 16S rRNA gene sequencing analysis, GC content, and AAI. The diversity was observed among the members of the genus *Bdellovibrio* thus suggesting the need to review the taxonomy of the genus *Bdellovibrio* in the nearest future. The pangenome analysis revealed that genomes of *Bdellovibrio* spp. have genes encoding different predation factors including signal transduction, hydrolytic, proteolytic, transport, and transport proteins that can help them survive as a bacterial predator. However, some factors such as hemolysin III and collagenase observed in the genomes need to be studied and characterized so that they will not have counterproductive effects when *Bdellovibrio* spp. are considered for applications as a biocontrol agent of pathogens in humans. Finally, *Bdellovibrio* sp. SKB1291214 have GEIs with atypical percent GC, *AmsF* protein, and a homologue of 4-diphosphocytidyl-2-C-methyl-D-erythritol kinase among its unique genes and an insertion of a 174 bp fragment in its *hit* locus region. These occurrences are presumptive indications of HGT in *Bdellovibrio* sp. SKB1291214.

## Figures and Tables

**Table 1:** Genomic features of *B. bacteriovorus* strains SSB218315 and *Bdellovibrio* sp. SKB1291214.

Genome information	*B. bacteriovorus* SSB218315	*Bdellovibrio* sp. SKB1291214
Chromosome size	3,769,537 bp	3,730,590 bp
Number of contigs	1	20
N_50_	—	199,513
GC content	50.5%	44.80%
Total RNA	41	40
Complete rRNAs	3	3
tRNAs	34	33
Noncoding RNAs	4	4
Total genes	3620	3677
Total CDS	3579	3637
Coding CDS	3536	3588
Phage	1 (incomplete)	1 (incomplete)
Genomic island (integrated method)	69	146

**Table 2:** Number of core genes, accessory genes, unique genes, and exclusively absent genes obtained from the pangenome analysis of 7 strains of *Bdellovibrio* spp.

Genome number	Strain	Number of core genes	Number of accessory genes	Number of unique genes	Number of exclusively absent genes
1	*B. bacteriovorus* HD100	795	2641	65	1
2	*B. bacteriovorus* strain Tiberius	795	2579	301	1
3	*B. bacteriovorus* W	795	1063	857	54
4	*B. bacteriovorus* 109J	795	2635	132	1
5	*B. bacteriovorus* SSB218315	795	2575	113	6
6	*B. exovorus* JSS	795	200	1572	725
7	*Bdellovibrio* sp. SKB1291214	795	1366	1343	27

## Supplementary Materials

Supplementary 1 Additional file 1a: genome analysis of *Bdellovibrio* spp. SKB1291214 and SSB218315 for the genes expressed by *B. bacteriovorus* HD100 during predation as reported by Lambert et al. [25]. Additional files 1b–f: presumed predation-enhancing factors in *Bdellovibrio* spp. SKB1291214 and SSB218315 predicted by RAST annotation server. (1b) Flagellar and chemotaxis factor. (1c) Transport and type IV pilus proteins. (1d) Stress response proteins. (1e) Factors associated with the production of degradative enzymes for the metabolism of molecules. (1f) Siderophores and defense factors. Additional file 1g: BLASTp analysis of genes annotated as hemolysin, collagenase, and RTX toxin in the genomes of *Bdellovibrio* spp.

Supplementary 2 Additional files 2a and b: predicted genomic islands in *Bdellovibrio* spp. SKB1291214 and SSB218315, respectively.

Supplementary 3 Additional file 3: pairwise evolutionary distance among *Bdellovibrio* spp.

Supplementary 4 Additional files 4a–f: pangenome analysis of *Bdellovibrio* spp. using the BPGA pipeline. (4a) The core genes identified in the *Bdellovibrio* spp. (4b) The power and exponential fit law to predict the nature of *Bdellovibrio* spp. pangenome as open or close. (4c–f) The unique genes identified in *Bdellovibrio* spp. SKB1291214, JSS, W, and SSB218315, respectively.

Supplementary 5 Additional 5a and b: multiple sequence alignment of the *Bd0108* and *Bd0109* genes of *hit* locus, respectively.
