# Supplementary material for: Whole-Genome Sequencing and Comparative Genome Analysis Provided Insight into the Predatory Features and Genetic Diversity of Two Bdellovibrio Species Isolated from Soil
Source: Int J Genomics. 2018 Apr 10;2018:9402073. doi: 10.1155/2018/9402073 (PMC5941755; doi:10.1155/2018/9402073)
Supplement: Supplementary 5 — Additional 5a and b: multiple sequence alignment of the Bd0108 and Bd0109 genes of hit locus, respectively. [file 9402073.f5.doc]

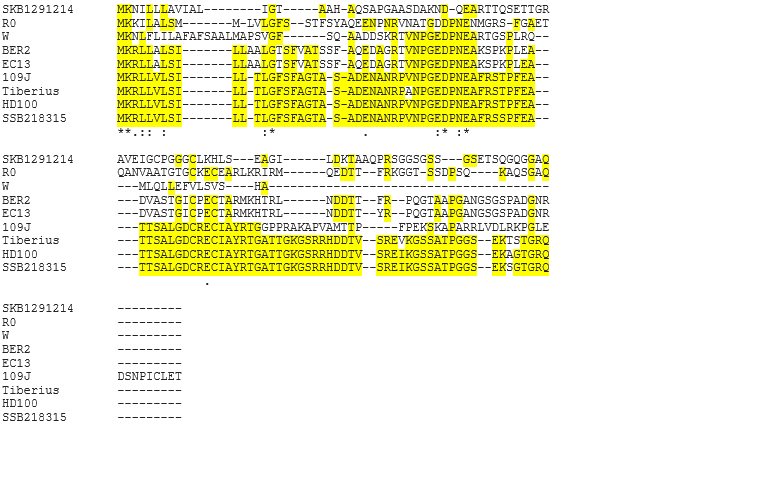


Additional file 5a. Multiple sequence alignment of the *Bd0108* gene of *hit* locus using CLUSTAL O (1.2.4)


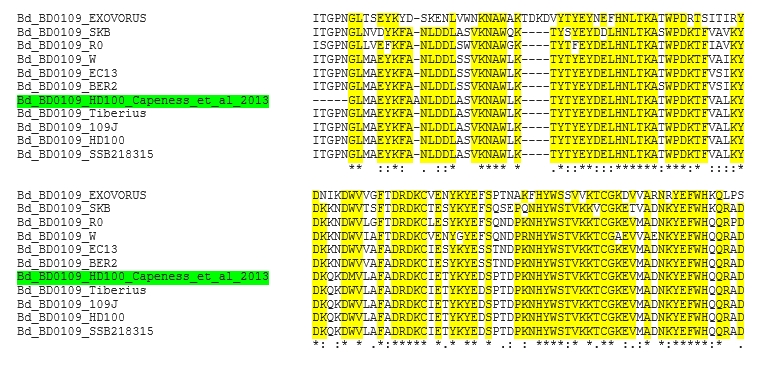


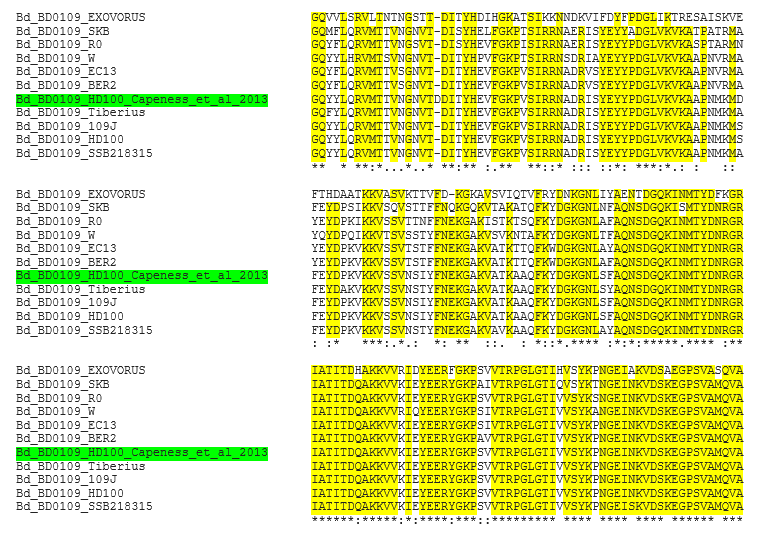


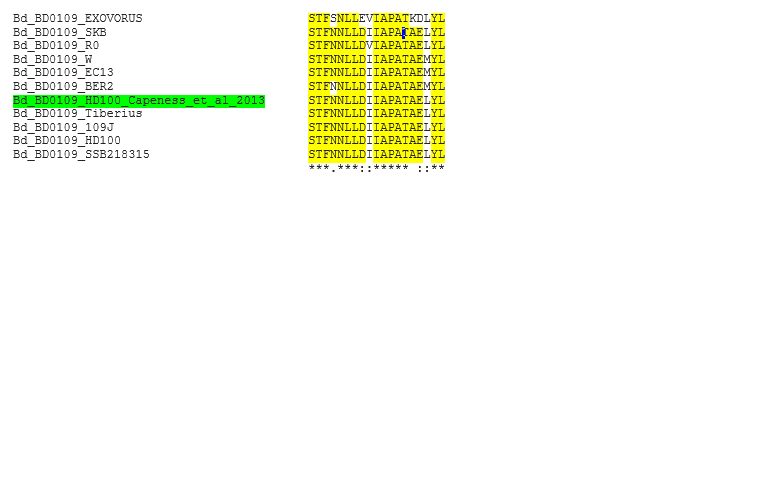


Additional file 5b. CLUSTAL O (1.2.4) multiple sequence alignment of Bd0109 gene of the *hit* locus. The amino acid sequence of *Bd0109* reported by Capeness et al., 2013 was aligned with Bd0109 region obtained from the other *Bdellovibrio* strains.
